# Removal of Ioban May Pull Bacteria to the Surface of the Skin: Lessons Learned

**DOI:** 10.51894/001c.12463

**Published:** 2020-06-08

**Authors:** Jacob Lytle, Jordan McCoy, Katie Smeltzer, Ryan Nelson

**Affiliations:** 1 Orthopedic Surgery Genesys Regional Medical Center

**Keywords:** shoulder surgery, cutibacterium acnes, orthopedics, iodine dressing

## Abstract

**CONTEXT:**

Cutibacterium acnes (C. Acnes, formerly known as Propionibacterium acnes) are slow growing, gram positive, anaerobic bacilli. C. acnes are found in many locations, both as part of normal skin flora, as well as a contaminant of environmental surfaces. These bacteria have been associated with prosthetic joint infections of the shoulder, and it has been challenging to prevent such infections for a variety of reasons. The purpose of this quality improvement project was to investigate whether the surgical adhesive dressing Ioban could pull subcutaneous C. acnes bacteria from the surgical field.

**METHODS:**

During this quality improvement project, a convenience sample of 16 adult volunteers were gathered from other residency departments and from medical students at our hospital. The volunteers were used to take samples from two sites on each shoulder. The shoulder sites were prepped and covered with iodine-impregnated dressings.

**RESULTS:**

There were 26 of 64 (40.6%) samples in the no Ioban group that grew C. acnes. The Ioban group had 37 of 64 (57.8%) samples found to be positive for C. acnes growth. During this project, we identified several key points that could be useful to future researchers working in community hospitals. We describe these lessons concerning ongoing communication with lab and research departments, offering volunteers compensation to participate, interacting with departments unaccustomed to research, and development of a clear methodology.

**CONCLUSIONS:**

This was the first time our department had conduct a project utilizing the laboratory as well as volunteers. This came with unforeseen challenges which caused significant time delays. We believe that by highlighting these lessons for future researchers, they might avoid such problems during project activities.

## INTRODUCTION

Cutibacterium acnes (C. Acnes, formerly known as Propionibacterium acnes) are slow growing, gram positive, anaerobic bacilli.^1,2^ C. acnes are found in many locations, both as part of normal skin flora, as well as a common contaminant during culturing due to shedding of epidermal cells.^2,3^ They have been associated with prosthetic joint infections of the shoulder, and it has been challenging to prevent such infections for a variety of reasons.^1,4–8^ The slow growth of C. acnes presents one challenge in the diagnosis of infection – if cultures are discarded in 3-5 days, the infection may not be detected.^1^ There is some evidence to suggest that cultures associated with a true infection, versus contamination (i.e., pre-infectious introduction of microorganisms), may be positive sooner in terms of time range (median of five days for a true infection vs. a median of nine days for contamination).^1^ Using polymerase chain reaction (PCR) for diagnosis may help overcome this challenge, and results are typically available within 24 hours.^1,3^

C. acnes has a propensity for the shoulder joint versus other joints, likely due to a higher level of colonization in that joint versus other joints (e.g., knee, hip).^1,9,10^ Younger, male patients are those at greater risk for deep infection after a primary shoulder arthroplasty, although other types of shoulder surgeries also have a risk for infection, with C. acnes being the most common infection-causing agent.^6,11^

In addition to being present on the dermal surface, C. acnes is also present in the sebaceous gland associated with hair follicles – hence males with more of these glands may be at greater risk.^4,10^ Topical agents may not be effective in penetrating below the dermal surface, making skin preparation more challenging with traditional germicidal agents for shoulder arthroplasties.^4^

C. acnes infections can often be insidious and asymptomatic, with no signs of inflammation or infection for late presenting cases.^5,6^ Most infections occur within two years after the surgical intervention, and one study showed the infection manifested a median of 210 days after the procedure.^5,6^ This can make diagnosis challenging, especially with the lack of symptoms.^5^

Patients with early infections can present in the typical manner and have signs of infection and inflammation. The sometimes-late onset and lack of symptoms can be addressed by considering multiple deep specimens for testing of C. acnes, even in patients undergoing revisions of shoulder arthroplasties years after the original procedure for mechanical problems, such as stiffness, impingement, or implant loosening.^6^

Traditional skin preparation and preoperative antibiotics have not uniformly been successful in preventing infection with C. acnes, posing another challenge to decreasing its incidence after shoulder arthroplasty.^7^ The skin preparation cannot achieve effectiveness in the sebaceous glands, which harbor huge numbers of C. acnes in each associated hair follicle.^4,7,8^ Benzoyl peroxide has historically been used to treat acne vulgaris which has been associated with C. acnes.^3,7^ A recent study by Kolakowski et al. that utilized benzoyl peroxide as an adjunct skin-cleansing agent prior to shoulder surgery, including arthroscopic surgery as well as shoulder arthroplasty, showed benzoyl peroxide may decrease the incidence of infection with C. acnes .^7^

Iodophor impregnated adhesive dressings, such as Ioban are commonly used in shoulder surgeries as a part of the sterile draping process.^12,13^ One study regarding hip procedures showed that using this type of drape caused a statistically significant reduction in the number of bacteria at the surgical site.^12^ It is often noted during the authors’ operative cases that there are grossly visible particles present on the adhesive surface of the dressing following removal of the dressing. This is of particular concern in shoulder surgeries, where C. acnes lies in the sebacious tissues of the deep dermis.^4,7,8^ Before the study, the authors theorized that the drape might actually aid in mobilizing the bacteria to the surface similar to over-the-counter adhesive acne treatments.^14^ For example, a common acne treatment uses hydroactive adhesive strips to pull bacterial plugs to the surface.^14^ This same effect has not been specifically studied in relation to surgical adhesive dressings.

### Purpose of Study

The purpose of this quality improvement project was to investigate whether the surgical adhesive dressing Ioban could pull subcutaneous C. acnes bacteria from the surgical field. Before the project, we had hypothesized that after standard skin prep of the shoulder and coverage and subsequent removal of Ioban, there would be bacterial growth in the cultures on the undersurface of the Ioban or the skin. Throughout the project, we faced multiple roadblocks while working in a community hospital with a smaller dedicated research department than is common at university centers. We learned valuable lessons throughout this process that may be helpful to review when starting such a project. In this paper, we discuss five key lessons learned during our project.

## METHODS

Volunteers were gathered from members of residency programs and medical students at the Genesys Medical Regional Medical Center in Grand Blanc, MI. Before data collection was begun, the authors’ institutional review board had given full approval for the project and subjects gave informed consent to participate in the project. A total of 16 subjects were gathered with eight male and eight female volunteers. Using each individual as their own comparison, we calculated that we would need 16 volunteers to see a significant difference of 20% change in bacterial load from before and after Ioban application. We used a comparative paired-sample students T test to determine the value. Inclusion criteria were any volunteer in either a residency program or rotating medical student who was 18 or older and willing to participate. Subjects were offered a $25 gift card for participation in the project which they would receive after signing the informed consent form. If at any point they wished to withdraw after consenting, they were informed they would still receive a gift card. No subjects withdrew from the study after signing consent forms. Funding for the gift cards and other expenses of this study was provided through Michigan State University through the 2018 Statewide Campus System Resident Scholarly Activity Support award program.

Exclusion criteria were any history of shoulder surgery, recent bacterial infection, or current antibiotic use. Subjects were administered a survey questionnaires developed for this project. Questionnaires were developed to include identify any exclusion criteria as stated above as well as general demographic data. Volunteers were instructed to come to the operative suite at our institution of Ascension Genesys Hospital and were given proper operative attire as dictated by hospital policy. They were brought back individually to an operating room that had been cleaned with the standard protocol before patient use at our institution.

Volunteers had both shoulders sampled and sent for culture. A total of 17 volunteers were recruited with one person dropping out due to their inability to participate on the day of sampling for a total of 16 volunteers with 32 shoulders sampled. There were two separate days of sampling with 13 volunteers participating on the first day and three on the second day.

All volunteers were brought back to the operating room individually and positioned on an operative bed in a seated position. Their bilateral shoulders were exposed using tape to retract scrub sleeves outside of the area to be prepped. Following this, each shoulder was prepped with a antimicrobial chlorhexidine (i.e., ChloraPrep) scrub stick, following standard protocol for our institution and a three-minute dry time was allowed. Iodine impregnated adhesive dressing, Ioban, was then applied to the shoulder in sterile fashion. After five minutes, the adhesive dressing was peeled back by one of the authors and using sterile gloves a second author would swab the shoulder sites. Each shoulder had four sites sampled: posterior shoulder skin, posterior on the adhesive surface of iodine dressing, anterior shoulder skin, and anterior adhesive surface of iodine dressing. A sterile back table was used to set up culture tubes and perform sampling and collection. In between each subject, the operating bed was cleaned to protect volunteers. Operating beds were cleansed between volunteers with Super Sani-Cloth germicidal wipes, this is the standard disinfectant wipe used to cleanse surfaces in room turnover at the authors institution.

Anaerobic culture tubes were used as C. acnes is anaerobic ^1,11^. Cultures were then sent to the lab at our institution and were grown in anaerobic culture on blood agar plates. Representative colonies from each plate grown anaerobically that appeared as a gram-negative rod on gram stain were sampled and confirmed as C. acnes with Indol testing. This method is the normal confirmatory process for C. acnes at our institution. There were two volunteers both male that also had control samples taken after shoulder was cleansed with chlorhexidine but before Ioban was placed. These eight samples were also sent to the lab for similar analysis. Our lab analysis was provided by one of the lab technologists at our institution who routinely performs culture analyses. Results from the cultures were entered into RedCap software for analysis.^15,16^ These analyses was performed by the research coordinator at our institution.

## RESULTS

Samples were collected from 16 volunteers with an equal number of eight males and eight females. Sampling of both shoulders and four sites per shoulder yielded eight samples per volunteer for a total of 128 samples. 64 of these sites were from the adhesive portion of the Ioban (Ioban) and 64 sites were from the skin of the volunteer (no Ioban). As seen in Table 1, the subgroups of volunteers were similar in regard to age and history of previous surgeries, history of diabetes, allergies, history of infection, steroid use, and antibiotic use. There were 26 of 64 (40.6%) samples in the no Ioban group grew C. acnes. The Ioban group had 37 of 64 (57.8%) samples were positive for C. acnes growth. This is a limited presentation of the results as the full review of the analysis and results will be described in a future separate paper.

**Table 1:** Bacteria growth of C. acnes on samples taken from Ioban sites versus samples taken from skin (no Ioban).

site_cat			Frequency	Percent	Valid Percent	Cumulative Percent
No Ioban	Valid	No	38	59.4	59.4	59.4
		Yes	26	40.6	40.6	100.0
		Total	64	100.0	100.0	
Ioban	Valid	No	27	42.2	42.2	42.2
		Yes	37	57.8	57.8	100.0
		Total	64	100.0	100.0	

## DISCUSSION

The primary purpose of this paper is to explicate the “lessons learned” from this project at our community hospital with limited prior experience with projects of this type.

### Lesson 1: Communication with lab departments and community research department (if available) is key

In our community-based hospital, we have a relatively small research department. When designing this project, we decided that we would need the lab department to plate and analyze our samples. Our initial plan was to have 128 samples, which was a large volume for our lab. In the early stages of our project development, it was a difficult hurdle to get the microbiology team engaged with our proposed research design. Due to space limitations, there was initial concern about the volume of samples required for this study, and whether or not the lab could support this in addition to normal patient samples.

At one point, it looked as if we would have to outsource our lab work - which would have been costly and perhaps unattainable due to the limited resources our small research department could provide. However, in working closely with our site research faculty, pathologists and lab administrators, we developed plans to break the plating down into four samples per plate to minimize resource utilization and accommodate all parties. This project activity took much longer than expected and our realizing this prior to our initial planning stage could have led to earlier involvement with lab managers and quicker turn around for our project results.

### Lesson 2: Offering compensation to volunteers is a significant boost for participation

When we were preparing for the project, we calculated that we would need a sample size of 16 volunteers using both shoulders. With 16 volunteers and four sites per shoulder, we estimated that we would require a total of 128 cultures. With a single day planned for sampling, it was difficult to find available volunteers at our center. HA pertinent hospital policy at our institution required training in operating area procedures in order to enter the operating theater. This required us to enroll only volunteers who had received training to enter the operating theater (i.e., residents and medical students), which vastly limited our pool of available volunteers.

Furthermore, medical students and residents typically have unpredictable, inflexible schedules, with very limited free time. Many were initially hesitant to participate due to the time commitment that was small, but not insignificant to the pool of potential volunteers. We decided to use our project funding to offer a $25 gift card to compensate all volunteers for their time. We also provided food and drink to the volunteers waiting to have sampling performed. There was a much higher willingness to participate after these changes were made, allowing us to reach our goal of 16 volunteers. We did have two volunteers drop out prior to completing the project. These volunteers still received their gift cards as all consented volunteers were told that they retained their right to withdraw from a project without losing their incentives. From our experience in this study using human volunteers in an experimental study, we found it very helpful to offer compensation for time and inconvenience in order to reach the numbers needed for sample size.

### Lesson 3: Communication with departments not accustomed to performing research can be challenging

During our project, we required access to supplies such as anaerobic culture tubes, Ioban dressings, sterile table covers, and access to an operating room. At our community hospital, this was not a typical request, and the number of supplies we needed was greater than the usual number supplied each day to the operating room suite. We attempted to communicate with operating nurse managers to obtain all necessary supplies, and learned that unforeseen errors could occur with uncommon requests and departments not accustomed to research projects. For example, on the day of sampling, the supplies requested did not match the supplies provided. We had requested 140 anaerobic culture tubes and instead received 140 unusable aerobic culture tubes.

This error was not identified until they were being opened, as the outer packaging did not make a distinction between aerobic or anaerobic. We were subsequently able to obtain enough anaerobic tubes from the surgical department to complete the majority of the sampling, but did have to reschedule some of the volunteers for a second day. This created an inconvenience for all parties involved. This oversight could have been avoided with more in-depth investigation of supplies being provided. This event emphasizes the importance of direct communication and verification with departments assisting with such a project.

### Lesson 4: Development of clear methodology for experimentation process

We planned to complete this project as a proof of concept that iodine-impregnated dressing could pull bacteria to the surface of the skin. To achieve this goal, we gathered volunteers to sample using sterile technique. We had scheduled individual time slots for each volunteer and had set up supplies that we believed would streamline the process. What we learned, however, was that it was more difficult than anticipated to manage volunteer arrival times and the sampling process.

During the study, we experienced complications related to volunteer arrival time, with some volunteers coming early and others arriving late. This altered our schedule and did result in one conflict where a volunteer had to withdraw from the study prior to sampling. Additionally, we did not initially take into consideration the turnover time that it would take in between each patient to clean the bed, set up supplies for the next sample, etc. Retrospectively, the process may have benefitted from a practice sampling session prior to the arrival of enrolled volunteers. Over time, we became much better at moving through sampling with each successive test subject. However this process still could have been made more efficient at the start of the project by including a preparation day with sample testing.

### Lesson 5: Know your research department

Although the research department at our institution was small, they were instrumental in allowing us to complete our project. Statistics can be a time-consuming part of research and errors in data entry can be devastating to any research project. When attempting to enter data manually, the research department was able to assist with transition from a simple spreadsheet into RedCap, which is the data entry and statistics tool used at our institution. This process would have taken much longer time without the aid of a research assistant who was better versed in the aims of the project. After completion of this project, it was obvious that there is a significant need to discuss goals and data collection needs with whomever would be performing the statistical analysis.

## CONCLUSIONS

In conclusion, this was the first time we had attempted such a project at this institution. Although a small number of previous residents in our department have collected project data collection, this was the first time the orthopedics department had used human volunteers for data collection. This study was a learning experience for the authors as well as for the research department members. In the future, we hope to perform more prospective experimental research, as the majority of the projects coming out of our department have been retrospective in nature. Ideally, this project prospective design will lead to a higher level of future evidence concerning this phenomenon and contribute to the orthopedic literature.

### Conflict of Interest

The authors declare no conflict of interest.
